# Associations Between Paraspinal Muscle Degeneration, Trunk Isokinetic Strengths, and Spinopelvic Alignment in Patients Undergoing Lumbar Fusion: A Cross-Sectional Study

**DOI:** 10.3390/jcm15155812

**Published:** 2026-07-24

**Authors:** Jiahua Feng, Fangyi Wei, Zhongning Xu, Jintao Ao, Tenghui Ge, Renxian Wang, Fangfang Duan, Jingye Wu, Zhao Lang, Ling Wang, Yuqing Sun

**Affiliations:** 1 Department of Spine Surgery, Beijing Jishuitan Hospital, Capital Medical University, Beijing 100035, China; 2025fengjh@mail.ccmu.edu.cn (J.F.); f_wei939@mail.ccmu.edu.cn (F.W.); lorchid_jst@163.com (Z.L.); 2 National Center for Orthopaedics, Beijing 100035, China; xuzhongning111@pku.edu.cn (Z.X.); 2311110591@pku.edu.cn (J.A.); 18811035655@163.com (T.G.); wrxpumc@126.com (R.W.); dffang1988@163.com (F.D.); wujingyewiseman@sina.com (J.W.); 3 Department of Spine Surgery, Beijing Jishuitan Hospital, Fourth Clinical College of Peking University, Beijing 100035, China; 4 JST Sarcopenia Research Centre, Beijing Research Institute of Traumatology and Orthopaedics, Beijing Jishuitan Hospital, Beijing 100035, China; 5 Clinical Epidemiology Research Center, Beijing Jishuitan Hospital, Capital Medical University, Beijing 100035, China; 6 Department of Radiology, Beijing Jishuitan Hospital, Capital Medical University, Beijing 100035, China

**Keywords:** degenerative lumbar disease, trunk isokinetic dynamometry, paraspinal muscle degeneration, spinopelvic alignment, multifidus, muscle strengths

## Abstract

**Background/Objectives:** Spinopelvic alignment represents the sagittal relationship between the lumbar spine and pelvis and is closely related to compensatory posture and disability in degenerative lumbar diseases. Previous studies have mainly focused on magnetic resonance imaging (MRI)-derived paraspinal muscle morphology, whereas evidence linking objective dynamic trunk strength to spinopelvic alignment remains limited. The present study aims to investigate the associations between spinopelvic alignment and muscle degeneration as assessed by MRI-based morphology and trunk isokinetic dynamometry. **Methods:** In this prospective cross-sectional study, patients undergoing lumbar fusion surgery for degenerative lumbar diseases at a tertiary center were consecutively enrolled. Peak trunk isokinetic torques were measured using the BioniX Sim3 Pro system. Preoperative paraspinal muscle degeneration and spinopelvic alignment were measured. **Results:** A total of 119 patients were analyzed (52.1% female). In the total sample, paraspinal muscle degeneration, isokinetic torques, and spinopelvic alignment showed statistically significant but generally weak associations. After adjustment for age, sex, and body mass index (BMI), rotation and flexion torques were associated with pelvic tilt, and multifidus functional cross-sectional area (MF-fCSA) was associated with lumbar lordosis (LL), pelvic tilt (PT), pelvic incidence-lumbar lordosis (PI-LL), and PI-LL mismatch. In the hypothesis-generating sex-stratified analyses, trunk isokinetic torques were associated with spinopelvic alignment in males but not females, whereas MF-fCSA was associated with spinopelvic alignment in both sexes. **Conclusions:** Paraspinal muscle morphology and trunk isokinetic strength were significantly associated with spinopelvic alignment, although the magnitudes of these associations were weak. These findings suggest that imaging-based muscle morphology and dynamic trunk muscle performance are associated with spinopelvic alignment but their implications should be interpreted with caution. Paraspinal muscle imaging and dynamic isokinetic strengths may provide complementary information in the assessment of patients with degenerative lumbar disease. Longitudinal studies are needed to determine their prognostic and clinical value.

## 1. Introduction

Paraspinal sarcopenia, characterized by reduced paraspinal muscle quantity and increased fatty infiltration, has been gaining attention in orthopedic research as a potentially modifiable risk factor for spinal degeneration, impaired function, and adverse clinical outcomes [1,2,3,4,5,6,7]. Degenerative lumbar diseases, including lumbar spinal stenosis, degenerative spondylolisthesis, and lumbar disc degeneration, are major causes of pain and disability in older adults and are frequently accompanied by deterioration of paraspinal muscle quality and alterations in spinopelvic alignment [8]. Spinopelvic alignment represents the geometric relationship between the lumbar spine and pelvis. Patients with degenerative spinal diseases (DSD) commonly develop spinopelvic malalignment characterized by reduced lumbar lordosis, increased pelvic retroversion, and PI-LL mismatch, which severely impair standing posture, walking ability, pain, disability, and quality of life [9,10]. Given the essential role of trunk and paraspinal muscles in spinal stability, the contributions of muscle degeneration to spinopelvic malalignment have attracted increasing research attention [6,11,12].

Most previous studies have evaluated paraspinal muscles using imaging-based morphologic parameters, isometric dynamometry, or endurance tests. Although MRI-based measurements such as cross-sectional area (CSA), fCSA, and fatty infiltration (FI) reflect structural characteristics of paraspinal muscles, these static measures provide limited information about actual muscle strength [1,13]. Trunk isokinetic dynamometry is a validated approach for assessing muscle strength [14,15] that allows quantitative assessment of dynamic muscle strength during rotation, extension, flexion, and lateral flexion movements [1,16]. Thus, combining MRI-based morphology with isokinetic dynamometry may provide complementary structural and functional information for preoperative assessment, rehabilitation planning, and postoperative management in patients undergoing lumbar fusion. Previous studies investigating associations between paraspinal muscle degeneration and spinopelvic alignment have largely focused on imaging paraspinal metrics [17,18,19,20]. However, studies integrating morphological and functional metrics for muscle assessment remain scarce. Evidence regarding associations between isokinetic trunk muscle strength and spinopelvic alignment also remains limited.

The aim of this study is to investigate the associations between spinopelvic alignment and muscle degeneration as assessed by imaging parameters and isokinetic dynamometry. Given the critical role of muscle degeneration in spinopelvic malalignment [6,21], we hypothesized that muscle degeneration would be positively associated with parameters of malalignment such as PT and PI-LL, and negatively related with LL and sacral slope (SS).

## 2. Materials and Methods

### 2.1. Population

**Figure 1:**
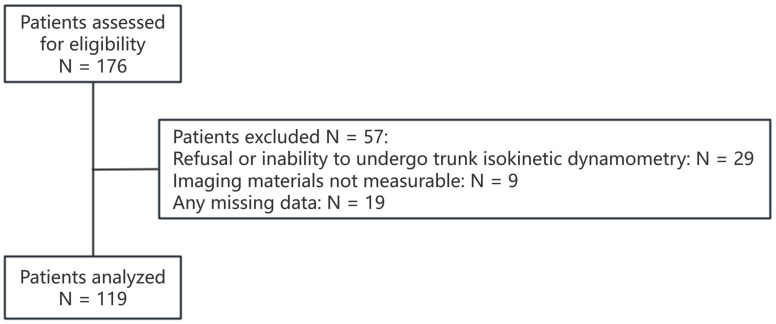
Study flowchart of patient inclusion and exclusion.

Patients undergoing lumbar fusion surgery for degenerative lumbar diseases at a tertiary medical center between June 2023 and June 2024 were prospectively and consecutively enrolled. During the study period, 176 patients were assessed for eligibility; 57 were excluded before final analysis because of refusal or inability to undergo trunk isokinetic dynamometry (*n* = 29), non-measurable imaging materials (*n* = 9), or missing data (*n* = 19). Written informed consent was obtained from all participants. Routine preoperative MRI and standing radiographs were obtained as part of standard clinical evaluation. No additional MRI or radiographic examination was performed for research purposes. Inclusion criteria were as follows: (I) age between 60 and 90 years; (II) main diagnosis of lumbar degenerative diseases; (III) signed informed consent; and (IV) complete imaging materials, with lateral lumbar radiographs including the measurable lumbar spine and both femoral heads. The exclusion criteria were as follows: (I) refusal or inability to undergo trunk isokinetic dynamometry; (II) Oswestry Disability Index (ODI) ≥ 80%; (III) significant spinal deformity (scoliosis with a Cobb angle greater than 20 degrees or lumbar spondylolisthesis higher than grade II); (IV) a history of lumbar trauma, lumbar surgery, or infectious diseases; (V) a history of neurological or neuromuscular diseases, autoimmune diseases, or tumors; and (VI) use of medications known to affect muscle structure or function. The study flowchart is shown in Figure 1.

### 2.2. Trunk Isokinetic Torque and Paraspinal Muscle Measurements

All participants underwent trunk isokinetic strength testing following a standard protocol using the BioniX Sim3 Pro device (Physiomed, Schnaittach, Germany) [22]. A testing velocity of 15°/s was selected to facilitate controlled trunk motion and maximal voluntary torque assessment in older symptomatic surgical candidates, with previous research supporting the reproducibility of measurements at this velocity [23]. After a 5-min walking warm-up, participants completed range-of-motion familiarization and low-intensity practice trials on the device before formal measurements. Participants then performed standing trunk isokinetic testing in rotation (ROT), extension (E), flexion (F), and lateral flexion (LF) at an angular velocity of 15°/s, with a 20-s rest between movements. Each movement was repeated five times, and standardized verbal encouragement was provided after each repetition. The participants were secured on the device via pelvic stabilization and instructed not to bend their knees during the movements. Peak torques for extension and flexion and average peak torques of both sides for rotation and lateral flexion were used for the analysis. Immediately before isokinetic testing, current back-pain and leg-pain intensities were recorded using separate 0–10 visual analogue scale (VAS) scales, and the higher of the two scores was used in the analysis. Oswestry Disability Index (ODI) was also assessed immediately before testing.

Paraspinal muscle morphology was evaluated on axial T2-weighted MRI at the L4/5 intervertebral disc level. Muscle segmentation and measurements were performed using ImageJ (version 1.54f; National Institutes of Health, Bethesda, MD, USA) by one orthopedic research fellow with 3 years of experience. The assessor was blinded to other relevant data including isokinetic torques and spinopelvic alignment. Regions of interest (ROI) were manually traced to delineate the multifidus (MF), erector spinae (ES), and psoas major (PS). Fatty area and fCSA were differentiated using a standardized grayscale thresholding method by the software. Fatty infiltration (FI) was calculated as fatty area/total area × 100%. All measurements were performed bilaterally, and the mean values were calculated for analysis. This method has been shown to have excellent intra- and inter-rater reliability in previous studies [24]. Thirty cases were randomly selected and re-assessed by the same assessor for intra-observer reliability and by another orthopedic research fellow with 3 years of experience for inter-observer reliability. The overall intra-observer intraclass correlation coefficient (ICC) was 0.978 (95% CI, 0.959–0.986); the inter-observer ICC was 0.933 (95% CI, 0.867–0.962).

**Figure 2:**
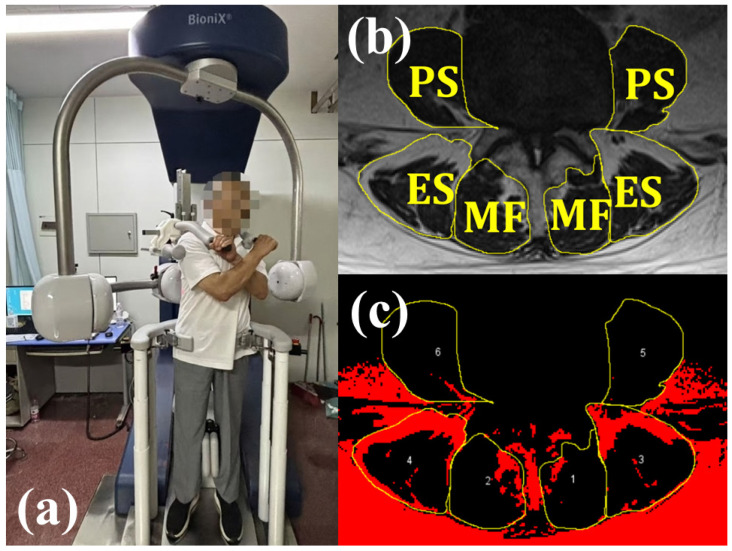
Illustration of a patient taking trunk isokinetic rotation torque measurement (**a**), the outline of multifidus (MF), erector spinae (ES) and psoas major (PS) at the L4/5 intervertebral disc level on axial T2-weighted MRI (**b**) and the corresponding binarized figure as displayed in the ImageJ interface (**c**).

An illustration of trunk isokinetic torque and paraspinal muscle measurement is shown in Figure 2.

### 2.3. Spinopelvic Alignment Measurements

**Figure 3:**
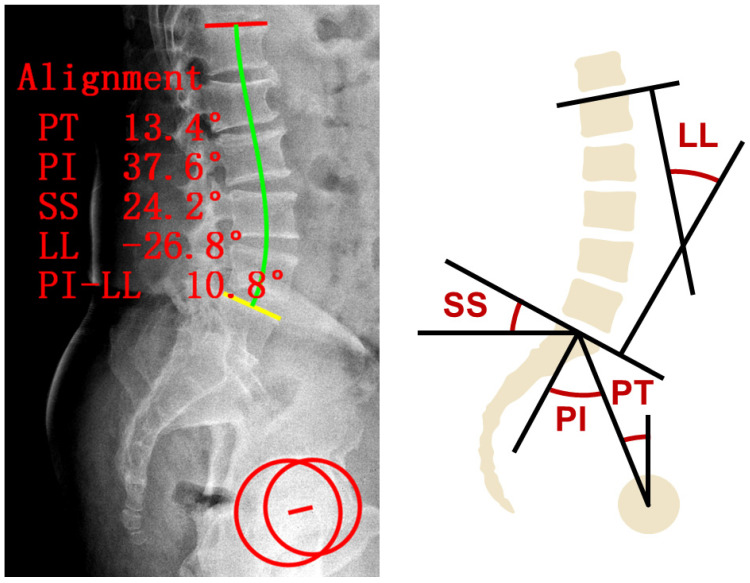
Spinopelvic alignment measurements, including lumbar lordosis (LL), sacral slope (SS), pelvic incidence (PI), and pelvic tilt (PT), shown on a lateral lumbar radiograph (**left**) and a schematic diagram (**right**). On the radiograph, the red and yellow lines indicate the superior endplates of L1 and S1, respectively, and the green arc represents the lumbar lordosis measurement. The red circles delineate the femoral heads used to determine the bicoxofemoral axis.

Spinopelvic parameters were measured on routine preoperative standing lateral radiographs including the entire lumbar spine and both femoral heads. No additional radiographs were obtained solely for this study. All measurements were performed by one orthopedic research fellow with 3 years of experience using Surgimap (Version 2.3.2.1; Nemaris Inc., New York, NY, USA). The evaluated parameters included LL, PT, pelvic incidence (PI), SS, PI-LL, and PI-LL mismatch, which was defined as PI-LL > 10°. The assessor was blinded to other data of the patients. The reliability of these measurement methods has been demonstrated in previous studies [25]. Thirty cases were randomly selected and re-assessed by the same assessor for intra-observer reliability and by another orthopedic research fellow with 3 years of experience for inter-observer reliability. The overall intra-observer ICC was 0.988 (95% CI, 0.982–0.992); the inter-observer ICC was 0.975 (95% CI, 0.966–0.982). An example of spinopelvic alignment measurements is shown in Figure 3.

### 2.4. Sample Size Considerations

No formal a priori hypothesis-driven sample-size calculation was performed. After completion of recruitment, the adequacy of the final sample relative to the complexity of the prespecified regression models was evaluated using commonly cited rules of thumb. The primary sample-size consideration was the adequacy of the final cohort for the planned multivariable regression analyses. To reduce model complexity and avoid overfitting, each primary regression model included one muscle-related predictor at a time, either a trunk isokinetic torque parameter or a paraspinal muscle imaging parameter, with adjustment for age, sex, and BMI. Therefore, each primary model included approximately four predictor parameters. According to Green’s rule of thumb for multiple regression, the required sample size is N ≥ 50 + 8m for testing the overall model and N ≥ 104 + m for testing individual predictors, where m is the number of predictors. For the primary models in the present study, m = 4, corresponding to minimum sample sizes of 82 and 108, respectively [26]. Also, based on the commonly used rule of 15–20 participants per predictor parameter for multivariable linear regression, approximately 60–80 participants would be required. The final analyzed sample of 119 patients exceeded these requirements. Sixty-four cases of PI-LL mismatch also resulted in 16 events per predictor parameter sufficient for the primary logistic regression [27]. By contrast, the sex-stratified analyses included 57 males and 62 females and were therefore treated as hypothesis-generating rather than formal subgroup analyses.

### 2.5. Statistical Analysis

Continuous variables were presented as mean ± standard deviation (SD) or median with interquartile range (IQR), according to distributional characteristics. Normality was assessed using the Shapiro–Wilk test and visual inspection of histograms and quantile–quantile (Q-Q) plots. Categorical variables were presented as number (percentage). Intra- and interobserver reliability of paraspinal muscle and spinopelvic measurements was assessed using single-measure, absolute-agreement intraclass correlation coefficients derived from two-way mixed- and random-effects models, respectively, with 95% confidence intervals. The overall ICC was calculated by pooling the standardized measurements across the evaluated parameters. Between-group differences were assessed using the independent-samples *t*-test, Welch’s *t*-test, Mann–Whitney U test, or Chi-square test, as appropriate. Spearman correlation analysis was used to examine associations between muscle-related parameters and spinopelvic alignment.

Multivariable linear regression analyses were performed for continuous spinopelvic outcomes, including LL, SS, PT, PI, and PI-LL. In the primary analyses, each trunk isokinetic torque parameter and each paraspinal muscle degeneration parameter was entered separately as an independent variable, with adjustment for age, sex, and body mass index (BMI). Multicollinearity was assessed using the variance inflation factor (VIF); maximum VIFs were lower than 3 in all models. For linear regression models, independence of residuals was evaluated using the Durbin–Watson statistic. Regression assumptions were assessed both numerically and graphically using residual-versus-fitted plots and Q-Q plots. When regression assumptions were violated, bias-corrected and accelerated (BCa) bootstrapping with 1000 resamples was applied. Binary logistic regression was performed for PI-LL mismatch. Each muscle-related predictor was entered separately, with adjustment for age, sex, and BMI in the overall cohort and for age and BMI in the sex-stratified analyses. Formal sex-by-muscle interaction models were not performed because the study was not powered for interaction testing; sex-stratified analyses were therefore considered hypothesis-generating. Statistical significance was defined as *p* < 0.05. All analyses were conducted using Python version 3.12.10.

## 3. Results

### 3.1. Demographic Characteristics and Spinopelvic Parameters

A total of 119 patients were analyzed, of which 62 were females. There were no significant differences in age, BMI, or ODI between the two sexes. VAS for maximum back or leg pain was higher in males (5.0 vs. 4.0, *p* = 0.012). Males had lower PT (15.5° vs. 22.8°, *p* = 0.005) and PI (44.8° vs. 49.6°, *p* = 0.024). There were no significant differences in LL, SS, PI-LL and percentage of PI-LL mismatch between the two sexes. Baseline demographic characteristics and spinopelvic parameters are shown in Table 1.

### 3.2. Trunk Isokinetic Torques and Paraspinal Muscle Degeneration

Males had higher trunk isokinetic torques than females in rotation, flexion, and lateral flexion (ROT: 24.3 vs. 19.3 Nm; F: 60.1 vs. 46.8 Nm; LF: 51.4 vs. 42.3 Nm; all *p* < 0.05). Extension torque was also higher in males, although the difference did not reach statistical significance (54.4 vs. 48.8 Nm, *p* = 0.069). Functional CSA-related parameters were higher in males (MF-fCSA: 1444.2 vs. 1030.3 mm^2^; ES-fCSA: 2412.5 vs. 1900.9 mm^2^; PS-fCSA: 2747.1 vs. 1711.3 mm^2^; all *p* < 0.001). PS-FI was lower in males (2.2% vs. 4.1%, *p* = 0.026), whereas MF-FI and ES-FI did not differ significantly between sexes. These results are shown in Table 2.

### 3.3. Correlations Between Trunk Isokinetic Torques, Paraspinal Muscle Degeneration, and Spinopelvic Alignment

Correlations between spinopelvic alignment and trunk isokinetic torques showed distinct sex-specific patterns, although the magnitudes of significant correlations were mostly weak to moderate. In males, rotation torque correlated with LL (r = 0.313, *p* < 0.05), SS (r = 0.275, *p* < 0.05), PT (r = −0.272, *p* < 0.05), and PI-LL (r = −0.267, *p* < 0.05). Extension torque correlated with LL (r = 0.265, *p* < 0.05), PT (r = −0.316, *p* < 0.05), and PI-LL (r = −0.303, *p* < 0.05), whereas lateral flexion torque correlated only with LL (r = 0.332, *p* < 0.05). No spinopelvic parameter correlated with flexion torque in males. In females, no significant correlations were found between trunk isokinetic torques and spinopelvic parameters.

Correlations between spinopelvic alignment and paraspinal muscle degeneration showed similar sex-specific patterns. In males, MF-fCSA correlated with LL (r = 0.370, *p* < 0.01), SS (r = 0.265, *p* < 0.05), PT (r = −0.261, *p* < 0.05), PI (r = −0.330, *p* < 0.05), and PI-LL mismatch (r = −0.318, *p* < 0.05). In females, MF-fCSA correlated only with LL (r = 0.284, *p* < 0.05), PI-LL (r = −0.335, *p* < 0.01), and PI-LL mismatch (r = −0.255, *p* < 0.05). No other fCSA- or FI-related parameter significantly correlated with spinopelvic alignment in either sex.

The correlation between trunk isokinetic torques and paraspinal muscle degeneration was also analyzed. In the overall cohort, trunk isokinetic torques were significantly correlated with all fCSA-related parameters (r = 0.227 to 0.368, all *p* < 0.05). Among FI-related parameters, MF-FI correlated with extension, flexion, and lateral flexion torques (r = −0.187 to −0.232, all *p* < 0.05). After sex stratification, different patterns were observed. In males, isokinetic torques correlated with fCSA-related parameters (r = 0.262 to 0.429, *p* < 0.05) depending on the comparisons, whereas no significant correlations were found in FI-related parameters. In females, no significant correlations were found between isokinetic torques and fCSA-related parameters, whereas MF-FI was significantly correlated with ROT, E, F, and LF torques (r = −0.268 to −0.331, *p* < 0.05). Full correlation results are shown in Supplemental Materials Tables S1–S3 (Tables, which demonstrate correlation results in the total, male, and female populations, respectively).

### 3.4. Regression Analysis of Spinopelvic and Muscle Parameters in the Overall Cohort

In the total population, after adjusting for age, sex, and BMI, ROT (b = −0.225, β = −0.201, *p* = 0.036) and F (b = −0.097, β = −0.223, *p* = 0.016) were associated with PT. Notably, the association between E and PT reached borderline significance (b = −0.068, *p* = 0.056). MF-fCSA was associated with LL (b = 0.015, β = 0.396, *p* < 0.001), PT (b = −0.010, β = −0.352, *p* = 0.004), PI-LL (b = −0.017, β = −0.416, *p* < 0.001), and PI-LL mismatch (b = −0.002, standardized OR = 0.470, *p* = 0.005). Other associations between trunk isokinetic torques or paraspinal muscle degeneration and spinopelvic alignment were not significant. Although statistically significant, the effect magnitudes were limited. Significant regression results in the total sample are shown in Table 3.

### 3.5. Hypothesis-Generating Sex-Stratified Analysis

Hypothesis-generating sex-stratified analysis revealed different trends between the two sexes. In males, multivariable regression analysis showed that SS was associated with ROT (b = 0.235, β = 0.367, *p* = 0.010) and LF (b = 0.126, β = 0.379, *p* = 0.008) torques. PT was associated with ROT (b = −0.363, β = −0.405, *p* = 0.003), E (b = −0.124, β = −0.358, *p* = 0.008), and F (b = −0.145, β = −0.375, *p* = 0.006) torques. PI-LL was associated with ROT torque (b = −0.415, β = −0.348, *p* = 0.014). LL, PI, and PI-LL mismatch were not associated with trunk isokinetic torques. In females, no significant associations were found between isokinetic torques and spinopelvic alignment. These sex-stratified findings should be regarded as hypothesis-generating. Significant regression results are shown in Table 4.

Multivariable regression demonstrated that MF-fCSA was negatively associated with PT (b = −0.009, β = −0.352, *p* = 0.013) and PI-LL (b = −0.012, β = −0.348, *p* = 0.016) in males. In females, MF-fCSA was positively associated with LL (b = 0.021, β = 0.341, *p* = 0.004) and negatively associated with PI-LL (b = −0.027, β = −0.435, *p* < 0.001) and PI-LL mismatch (b = −0.003, standardized OR = 0.500, *p* = 0.031). No other imaging parameters for paraspinal muscle degeneration were associated with spinopelvic alignment. Significant regression results are shown in Table 5.

## 4. Discussion

The present study investigated the associations between paraspinal muscle degeneration, trunk isokinetic strength, and spinopelvic alignment in patients undergoing lumbar fusion for degenerative lumbar diseases. By integrating MRI-based paraspinal muscle parameters and objective isokinetic strength measurements, our findings may provide insights into the muscle–alignment relationship and inspire future studies on preoperative or postoperative rehabilitation, risk stratification, and postoperative management in patients undergoing lumbar fusion. In the overall cohort, rotation and flexion torques were associated with lower PT after adjustment for age, sex, and BMI, while extension torque showed a borderline association in the same direction. In addition, MF-fCSA was consistently associated with more favorable spinopelvic alignment, including greater LL and lower PT, PI-LL, and PI-LL mismatch. These findings suggest that both muscle morphology and dynamic trunk muscle performance are related to spinopelvic alignment in the study population.

The magnitude of these associations was limited, and the results should be interpreted with caution. Statistically significant associations do not necessarily imply clinically meaningful relationships. Spinopelvic alignment in patients with degenerative lumbar diseases is associated with multiple factors, including pelvic morphology, vertebral, disc and facet degeneration, age, sex, BMI, hip and lower-extremity compensation, pain, and muscle degeneration [8,28]. Therefore, paraspinal muscle degeneration or trunk strength alone should not be expected to explain a large proportion of sagittal alignment variation. The presence of significant associations after adjustment for age, sex, and BMI suggests that muscle-related parameters may still be relevant to spinopelvic alignment. Rather than indicating a dominant causal effect, our findings suggest only a potential and partial role of muscle degeneration in maintaining spinopelvic alignment.

These results are consistent with the concept that the spinal column is stabilized through passive, active, and neural control subsystems [29]. The paraspinal muscles, especially the multifidus, are an important part of the active stabilizing subsystem. In the present study, MF-fCSA was the only imaging parameter consistently associated with spinopelvic alignment in multivariable regression. This finding supports the functional importance of the multifidus in maintaining lumbar lordosis and limiting compensatory pelvic retroversion [21,30]. Moreover, as a critical stabilizing muscle of the spine, the multifidus has been shown to play an important role in functional recovery, surgical outcomes and reoperation [31]. Our results are consistent with previous studies and further demonstrate the essential role of multifidus in lumbar degenerative diseases. Thus, assessment of MF-fCSA may have potential value for risk stratification, postoperative management, prehabilitation and rehabilitation planning since muscle degeneration is a potentially modifiable risk factor [32,33,34]. However, MF-fCSA should not be used as a standalone marker of alignment risk because the observed associations were weak and sagittal alignment is multifactorial. It should be noted that the lack of significant associations for other FI- or fCSA-related parameters does not preclude the importance of these parameters. Muscle degeneration is heterogeneous across spinal levels and muscle groups, and a single axial measurement at L4/5 may not fully capture global paraspinal degeneration. Moreover, fCSA may be more sensitive than total CSA or FI alone because it partially integrates muscle quantity and quality by estimating the contractile component of the muscle.

Our results have also demonstrated associations between trunk isokinetic torques and PT. PT is commonly interpreted as a compensatory parameter reflecting pelvic retroversion in response to sagittal malalignment. In the total sample, higher rotation and flexion torques were associated with lower PT, while extension torque showed a borderline association. The significant associations involving rotation and flexion torques should not be interpreted as evidence that these movements independently determine sagittal alignment. Rotation torque reflects the coordinated function of both abdominal wall muscles and paraspinal muscles, and both groups contribute to trunk stability [11,35,36,37,38,39]. Our results also suggested a protective association between flexion torque and PT. Although flexion strength acts in the opposite direction to lumbar extension, which is the most direct functional correlate of the posterior paraspinal muscles, flexion torque may also reflect overall trunk muscle reserve in this cohort. The baseline data suggested that flexion and extension torques were broadly comparable in magnitude, although statistical significance for difference was not tested. Therefore, higher flexion torque may not represent an isolated flexor effect, but rather a marker of better coordinated trunk strength, including preserved antagonist extensor function. It should also be noted that in symptomatic patients undergoing lumbar fusion, extension movements may be more affected by pain or fear of movement [40]. In addition, although pelvic stabilization was applied during isokinetic testing, compensatory movement through the pelvis and hips may not have been completely eliminated, which could attenuate the measured association between extension torque and sagittal alignment.

Hypothesis-generating sex-stratified analyses suggested that the associations between trunk isokinetic torques and spinopelvic alignment were more evident in males than in females. However, the absence of significant associations in females should not be overinterpreted. It should be noted that after stratification, the limited sample size is not sufficiently powered to detect significant associations, and no formal sex-by-muscle interaction analysis was performed. The results from sex-stratified analysis should only be considered hypothesis-generating. Biological and morphological differences may have contributed to these results. Females in the present cohort had generally lower trunk torques and smaller fCSA-related parameters, and showed higher PS-FI. In females, MF-FI rather than fCSA-related parameters was correlated with trunk isokinetic torques, suggesting that muscle quality may be more closely related to functional impairment in this subgroup. Recent evidence also suggests that paraspinal fatty infiltration may relate to clinical outcomes in a nonlinear or threshold-like manner [41]. If fatty infiltration exceeds a certain level, the force-generating capacity of the preserved muscle may become globally impaired, resulting in reduced strength and a weaker observable association between strength and alignment. This may partly explain why structural degeneration remained associated with alignment in females, whereas dynamic torque parameters did not reach significance. Future studies with larger samples should formally test sex interactions and nonlinear associations between FI, fCSA, muscle strength, and spinopelvic alignment.

This study has several limitations. First, the cross-sectional design precludes causal inference and the absence of longitudinal follow-up prevents evaluation of prognostic value for postoperative outcomes. Second, no formal hypothesis-driven a priori power calculation was performed because this was an exploratory cross-sectional study based on consecutive eligible patients during the study period. Nevertheless, as described in Section 2.4, the adequacy of the final analyzed sample was evaluated according to Green’s rule of thumb and the complexity of the prespecified multivariable regression models. However, it should be noted that the sex-stratified analyses were hypothesis-generating and may have been underpowered. Third, the observed associations were modest and may be influenced by unmeasured or incompletely measured confounders such as pain inhibition, duration of symptoms, fear of movement, neurological symptoms, physical activity level, medication use, hip function, and lower-extremity compensation. Fourth, multiple correlation and regression analyses were performed without formal multiplicity correction, which may increase the risk of type I error. Therefore, the findings should be interpreted according to their consistency and effect magnitude rather than *p* values alone. Fifth, radiographic assessment was limited to spinopelvic parameters and did not include full-spine sagittal alignment or lower-extremity alignment. Sixth, muscle assessment was limited to trunk isokinetic torques and MRI-based measurements at a single L4/5 level; multilevel or three-dimensional muscle assessment, as well as evaluation of abdominal, hip, and lower-extremity muscles, may provide a more comprehensive understanding of muscle-alignment relationships. Finally, the study included only preoperative patients who were able to complete trunk isokinetic testing; therefore, the results may not be generalizable to patients with more severe disability or to nonsurgical populations.

In summary, this study showed weak but significant associations between trunk muscle strengths, multifidus morphology, and spinopelvic alignment in patients undergoing lumbar fusion. MF-fCSA was the most consistent imaging-based parameter associated with alignment, whereas rotation and flexion torques were associated with lower PT and extension torque only showed a borderline association. These findings support the relevance of paraspinal and trunk musculature in sagittal alignment, while also indicating that muscle-related parameters represent only one part of a multifactorial biomechanical system for alignment. Functional assessment of lumbar musculature has been increasingly discussed in rehabilitation research [34], and our findings support the concept that structural and functional muscle assessment may be complementary. Longitudinal studies incorporating multilevel muscle imaging, comprehensive functional testing, pain-related variables, rehabilitation interventions, and postoperative outcomes are needed to clarify the clinical significance of these associations.

## 5. Conclusions

In patients undergoing lumbar fusion for degenerative lumbar diseases, paraspinal muscle morphology and trunk isokinetic strength were weakly but significantly associated with spinopelvic alignment. MF-fCSA showed the most consistent associations with spinopelvic parameters, while rotation and flexion torques were associated with lower PT. These findings suggest that imaging-based muscle morphology and dynamic trunk muscle performance are associated with spinopelvic alignment and may provide complementary information during the assessment of patients with degenerative lumbar disease. Longitudinal studies are needed to determine their prognostic and clinical value. Hypothesis-generating sex-stratified findings should be interpreted cautiously and confirmed in larger longitudinal studies.

## Figures and Tables

**Table 1:** Baseline characteristics and spinopelvic parameters of the study population.

Variables	Total *n* = 119	Males *n* = 57	Females *n* = 62	*p*-Value
Age, years	68.0 (64.0–71.0)	66.0 (63.0–70.0)	70.0 (65.0–72.0)	0.076
BMI, kg/m^2^	26.2 ± 3.3	26.1 ± 3.2	26.3 ± 3.4	0.743
ODI, %	44.1 ± 16.6	41.1 ± 15.2	47.0 ± 17.4	0.053
VAS	4.0 (3.0–6.0)	5.0 (4.0–6.0)	4.0 (3.0–5.0)	**0.01** **2**
LL, °	33.8 ± 13.2	33.5 ± 11.0	34.2 ± 15.1	0.752
SS, °	26.6 ± 8.6	26.1 ± 7.4	27.1 ± 9.7	0.558
PT, °	20.2 (13.5–28.0)	15.5 (13.0–22.8)	22.8 (16.9–30.1)	**0.005**
PI, °	47.3 ± 12.0	44.8 ± 9.3	49.6 ± 13.7	**0.024**
PI-LL, °	13.5 ± 15.0	11.3 ± 13.8	15.4 ± 15.9	0.136
PI-LL mismatch	64 (53.8%)	26 (45.6%)	38 (61.3%)	0.126

Values are presented as mean ± standard deviation, median (interquartile range), or n (%), as appropriate. *p* values indicate comparisons between males and females. Bold *p* values indicate statistical significance. Abbreviations: BMI, body mass index; ODI, Oswestry Disability Index; VAS, visual analogue scale; LL, lumbar lordosis; SS, sacral slope; PT, pelvic tilt; PI, pelvic incidence.

**Table 2:** Trunk isokinetic torques and paraspinal muscle degeneration of the study population.

Variables	Total *n* = 119	Males *n* = 57	Females *n* = 62	*p*-Value
ROT, Nm	21.9 (15.8–27.6)	24.3 (16.3–31.5)	19.3 (15.3–26.1)	**0.038**
E, Nm	51.6 (36.0–72.5)	54.4 (39.6–77.1)	48.8 (29.3–68.4)	0.069
F, Nm	53.2 ± 25.7	60.1 ± 26.9	46.8 ± 22.9	**0.004**
LF, Nm	46.7 ± 19.4	51.4 ± 22.4	42.3 ± 15.2	**0.012**
MF-fCSA, mm^2^	1228.5 ± 387.3	1444.2 ± 396.8	1030.3 ± 249.4	**<0.001**
ES-fCSA, mm^2^	2050.1 (1736.8–2481.5)	2412.5 (2050.1–2853.7)	1900.9 (1584.2–2073.9)	**<0.001**
PS-fCSA, mm^2^	2268.1 (1709.6–2713.5)	2747.1 (2429.6–3012.1)	1711.3 (1501.5–2032.2)	**<0.001**
MF-FI, %	20.7 (14.0–26.6)	21.0 (15.3–27.4)	20.4 (13.1–25.3)	0.436
ES-FI, %	30.8 (23.4–37.3)	31.9 (23.2–38.2)	30.3 (23.6–35.0)	0.764
PS-FI, %	3.3 (1.2–5.8)	2.2 (0.9–4.3)	4.1 (2.0–6.3)	**0.026**

Values are presented as mean ± standard deviation, median (interquartile range), or n (%), as appropriate. *p* values indicate comparisons between males and females. Bold *p* values indicate statistical significance. Abbreviations: ROT, rotation; E, extension; F, flexion; LF, lateral flexion; MF, multifidus; ES, erector spinae; PS, psoas major; fCSA, functional cross-sectional area; FI, fatty infiltration.

**Table 3:** Significant multivariable regression findings in the overall cohort.

Significant Predictor	Outcome	Effect Estimate (95% CI)	*p*-Value for Predictor
**Isokinetic Torques**			
ROT torque	PT	b = −0.225 (95% CI: −0.419 to −0.003) β = −0.201 (95% CI: −0.369 to −0.004) aR^2^ = 0.062	**0.036**
F torque	PT	b = −0.097 (95% CI: −0.178 to −0.010) β = −0.223 (95% CI: −0.388 to −0.022) aR^2^ = 0.069	**0.016**
**Paraspinal Imaging**			
MF-fCSA	LL	b = 0.015 (95% CI: 0.008 to 0.022) β = 0.396 (95% CI: 0.229 to 0.561) aR^2^ = 0.075	**<0.001**
	PT	b = −0.010 (95% CI: −0.016 to −0.003) β = −0.352 (95% CI: −0.525 to −0.156) aR^2^ = 0.080	**0.004**
	PI-LL	b = −0.017 (95% CI: −0.026 to −0.008) β = −0.416 (95% CI: −0.597 to −0.195) aR^2^ = 0.103	**<0.001**
	PI-LL mismatch	b = −0.002 (95% CI: −0.003 to −0.001) Standardized OR = 0.470 (95% CI: 0.277 to 0.797) Pseudo-R^2^ = 0.085	**0.005**

Values are reported as the unstandardized coefficient b (95% CI), standardized coefficient β (95% CI), standardized OR (95% CI), adjusted R^2^ (aR^2^) and pseudo-R^2^. All models were adjusted for age, sex, and BMI. Bold *p*-values indicate statistical significance. Abbreviations: CI, confidence interval; OR, odds ratio; F, flexion; LL, lumbar lordosis; MF-fCSA, multifidus functional cross-sectional area; PI-LL, pelvic incidence minus lumbar lordosis; PT, pelvic tilt; ROT, rotation.

**Table 4:** Significant associations between spinopelvic parameters and trunk isokinetic torques in regression analyses.

Significant Predictor	Outcome	Effect Estimate	*p*-Value for Predictor
**Males**			
ROT torque	SS	b = 0.235 (95% CI: 0.059 to 0.411)	**0.010**
		β = 0.367 (95% CI: 0.092 to 0.642)	
		aR^2^ = 0.101	
LF torque	SS	b = 0.126 (95% CI: 0.034 to 0.217)	**0.008**
		β = 0.379 (95% CI: 0.103 to 0.655)	
		aR^2^ = 0.107	
ROT torque	PT	b = −0.363 (95% CI: −0.599 to −0.127)	**0.003**
		β = −0.405 (95% CI: −0.668 to −0.141)	
		aR^2^ = 0.173	
E torque	PT	b = −0.124 (95% CI: −0.215 to −0.035)	**0.008**
		β = −0.358 (95% CI: −0.617 to −0.099)	
		aR^2^ = 0.149	
F torque	PT	b = −0.145 (95% CI: −0.247 to −0.043)	**0.006**
		β = −0.375 (95% CI: −0.639 to −0.111)	
		aR^2^ = 0.155	
ROT torque	PI-LL	b = −0.415 (95% CI: −0.744 to −0.086)	**0.014**
		β = −0.348 (95% CI: −0.625 to −0.072)	
		aR^2^ = 0.093	
**Females**			
No significant associations			

Only significant regression results are shown. Values are unstandardized coefficients (b) and standardized coefficients (β) with 95% CIs. Linear models report adjusted R^2^. All models were adjusted for age and BMI. Bold *p*-values indicate statistical significance. Abbreviations: b, regression coefficient; β, standardized regression coefficient; LL, lumbar lordosis; PT, pelvic tilt; PI, pelvic incidence; SS, sacral slope; ROT, rotation; E, extension; F, flexion; LF, lateral flexion.

**Table 5:** Significant associations between spinopelvic parameters and paraspinal muscle degeneration in regression analyses.

Significant Predictor	Outcome	Effect Estimate	*p*-Value for Predictor
**Males**			
MF-fCSA	PT	b = −0.009 (95% CI: −0.016 to −0.002)	**0.013**
		β = −0.352 (95% CI: −0.626 to −0.079)	
		aR^2^ = 0.135	
	PI-LL	b = −0.012 (95% CI: −0.022 to −0.002)	**0.016**
		β = −0.348 (95% CI: −0.628 to −0.068)	
		aR^2^ = 0.090	
**Females**			
MF-fCSA	LL	b = 0.021 (95% CI: 0.006 to 0.036)	**0.004**
		β = 0.341 (95% CI: 0.092 to 0.591)	
		aR^2^ = 0.081	
	PI-LL	b = −0.027 (95% CI: −0.043 to −0.013)	**<0.001**
		β = −0.435 (95% CI: −0.672 to −0.198)	
		aR^2^ = 0.169	
	PI-LL mismatch	b = −0.003 (95% CI: −0.005 to −0.000)	**0.031**
		Standardized OR = 0.500 (95% CI: 0.266 to 0.937)	
		Pseudo-R^2^ = 0.104	

Regression models were adjusted for age and BMI. Only significant regression results are shown. These hypothesis-generating analyses were not intended for formal subgroup inference. Bold *p*-values indicate statistical significance. Abbreviations: b, regression coefficient; β, standardized regression coefficient; LL, lumbar lordosis; PT, pelvic tilt; PI, pelvic incidence; MF, multifidus; fCSA, functional cross-sectional area.

## Data Availability

The data presented in this study are available from the corresponding author upon reasonable request. The data are not publicly available due to patient privacy and institutional ethical restrictions.

## Supplementary Materials

The following supporting information can be downloaded at https://www.mdpi.com/article/10.3390/jcm15155812/s1. Table S1: Correlation analysis between trunk isokinetic torques, paraspinal muscle degeneration, and spinopelvic alignment in the total population; Table S2: Correlation analysis between trunk isokinetic torques, paraspinal muscle degeneration, and spinopelvic alignment in males; Table S3: Correlation analysis between trunk isokinetic torques, paraspinal muscle degeneration, and spinopelvic alignment in females.

## Abbreviations

The following abbreviations are used in this manuscript:

DSD	Degenerative spinal diseases
ODI	Oswestry Disability Index
BMI	Body mass index
MRI	Magnetic resonance imaging
ROT	Rotation
E	Extension
F	Flexion
LF	Lateral flexion
MF	Multifidus
ES	Erector spinae
PS	Psoas major
CSA	Cross-sectional area
fCSA	Functional cross-sectional area
FI	Fatty infiltration
CI	Confidence interval
IQR	Interquartile range
OR	Odds ratio
SD	Standard deviation
ICC	Intraclass correlation coefficient
ROI	Regions of interest
LL	Lumbar lordosis
PT	Pelvic tilt
PI	Pelvic incidence
SS	Sacral slope
PI-LL	Pelvic incidence minus lumbar lordosis
VAS	Visual analogue scale
VIF	Variance inflation factor
BCa	Bias-corrected and accelerated
