# Supplementary material for: Associations Between Paraspinal Muscle Degeneration, Trunk Isokinetic Strengths, and Spinopelvic Alignment in Patients Undergoing Lumbar Fusion: A Cross-Sectional Study
Source: J Clin Med. 2026 Jul 24;15(15):5812. doi: 10.3390/jcm15155812 (PMC13466865; doi:10.3390/jcm15155812)
Supplement: Supplementary file 1 [file jcm-15-05812-s001.zip › Supplementary_Material Table_S3.pdf]

Table S3. Correlation analysis between trunk isokinetic torques, paraspinal muscle degeneration, and spinopelvic alignment in females.

	Age	BMI	ODI	mismatch	LL	SS	PT	PI	PI–LL	ROT torque	E torque	F torque	LF torque	VAS	MF-fCSA	ES-fCSA	PS-fCSA	MF-FI	ES-FI	PS-FI
**Age**	1.000	-0.006	0.207	0.028	0.089	0.216	-0.045	0.136	0.045	-0.303*	-0.263*	-0.276*	-0.143	0.101	-0.041	-0.058	-0.003	0.488***	0.266*	-0.107
**BMI**	-0.006	1.000	-0.079	-0.194	0.006	0.092	-0.073	-0.084	-0.098	0.255*	0.133	0.163	0.195	-0.118	0.003	0.247	0.361**	-0.154	-0.065	-0.041
**ODI**	0.207	-0.079	1.000	0.080	-0.051	0.086	-0.029	0.064	0.122	-0.356**	-0.370**	-0.418***	-0.447***	0.465***	-0.108	-0.211	-0.008	0.172	-0.157	-0.017
**mismatch**	0.028	-0.194	0.080	1.000	-0.457***	-0.180	0.446***	0.359**	0.844***	-0.224	-0.179	-0.116	-0.168	0.003	-0.255*	-0.152	-0.239	-0.105	0.015	0.117
**LL**	0.089	0.006	-0.051	-0.457***	1.000	0.795***	-0.143	0.408**	-0.591***	0.092	0.068	-0.049	0.025	0.239	0.284*	0.055	0.061	0.034	0.037	0.101
**SS**	0.216	0.092	0.086	-0.180	0.795***	1.000	-0.138	0.573***	-0.258*	0.105	0.030	-0.073	-0.012	0.326**	0.089	0.004	-0.130	-0.001	-0.044	0.124
**PT**	-0.045	-0.073	-0.029	0.446***	-0.143	-0.138	1.000	0.591***	0.642***	-0.105	-0.119	-0.196	-0.092	-0.003	-0.230	-0.092	-0.035	-0.065	-0.045	-0.042
**PI**	0.136	-0.084	0.064	0.359**	0.408**	0.573***	0.591***	1.000	0.430***	0.019	-0.043	-0.230	-0.089	0.286*	-0.129	-0.149	-0.169	0.014	-0.048	0.150
**PI–LL**	0.045	-0.098	0.122	0.844***	-0.591***	-0.258*	0.642***	0.430***	1.000	-0.173	-0.146	-0.169	-0.148	0.014	-0.335**	-0.154	-0.201	-0.039	-0.024	0.040
**ROT torque**	-0.303*	0.255*	-0.356**	-0.224	0.092	0.105	-0.105	0.019	-0.173	1.000	0.788***	0.695***	0.667***	-0.193	0.094	0.128	0.102	-0.331**	-0.118	0.039
**E torque**	-0.263*	0.133	-0.370**	-0.179	0.068	0.030	-0.119	-0.043	-0.146	0.788***	1.000	0.747***	0.625***	-0.025	0.110	0.113	0.177	-0.299*	-0.206	0.023
**F torque**	-0.276*	0.163	-0.418***	-0.116	-0.049	-0.073	-0.196	-0.230	-0.169	0.695***	0.747***	1.000	0.740***	-0.277*	0.093	0.150	0.092	-0.328**	-0.106	0.122
**LF torque**	-0.143	0.195	-0.447***	-0.168	0.025	-0.012	-0.092	-0.089	-0.148	0.667***	0.625***	0.740***	1.000	-0.387**	0.089	0.177	0.036	-0.268*	-0.056	0.158
**VAS**	0.101	-0.118	0.465***	0.003	0.239	0.326**	-0.003	0.286*	0.014	-0.193	-0.025	-0.277*	-0.387**	1.000	-0.085	-0.094	-0.065	0.147	-0.355**	0.040
**MF-fCSA**	-0.041	0.003	-0.108	-0.255*	0.284*	0.089	-0.230	-0.129	-0.335**	0.094	0.110	0.093	0.089	-0.085	1.000	0.206	0.210	-0.055	0.077	-0.234
**ES-fCSA**	-0.058	0.247	-0.211	-0.152	0.055	0.004	-0.092	-0.149	-0.154	0.128	0.113	0.150	0.177	-0.094	0.206	1.000	0.117	0.016	0.073	-0.104
**PS-fCSA**	-0.003	0.361**	-0.008	-0.239	0.061	-0.130	-0.035	-0.169	-0.201	0.102	0.177	0.092	0.036	-0.065	0.210	0.117	1.000	0.006	0.044	-0.266*
**MF-FI**	0.488***	-0.154	0.172	-0.105	0.034	-0.001	-0.065	0.014	-0.039	-0.331**	-0.299*	-0.328**	-0.268*	0.147	-0.055	0.016	0.006	1.000	0.539***	0.076
**ES-FI**	0.266*	-0.065	-0.157	0.015	0.037	-0.044	-0.045	-0.048	-0.024	-0.118	-0.206	-0.106	-0.056	-0.355**	0.077	0.073	0.044	0.539***	1.000	-0.008
**PS-FI**	-0.107	-0.041	-0.017	0.117	0.101	0.124	-0.042	0.150	0.040	0.039	0.023	0.122	0.158	0.040	-0.234	-0.104	-0.266*	0.076	-0.008	1.000

Values are Spearman correlation coefficients. * $p < 0.05$; ** $p < 0.01$; *** $p < 0.001$. PI–LL mismatch is defined as PI–LL > 10°. The matrix presents correlation coefficients together with their statistical significance.

Abbreviations: BMI, body mass index; VAS, visual analogue scale; LL, lumbar lordosis; SS, sacral slope; PT, pelvic tilt; PI, pelvic incidence; PI–LL, pelvic incidence minus lumbar lordosis; PI–LL mismatch, PI–LL > 10°; ROT torque, rotation torque; E torque, extension torque; F torque, flexion torque; LF torque, lateral flexion torque; ODI, Oswestry Disability Index; MF-fCSA, multifidus functional cross-sectional area; ES-fCSA, erector spinae functional cross-sectional area; PS-fCSA, psoas functional cross-sectional area; MF-FI, multifidus fatty infiltration; ES-FI, erector spinae fatty infiltration; PS-FI, psoas fatty infiltration.
