# Supplementary material for: Associations Between Paraspinal Muscle Degeneration, Trunk Isokinetic Strengths, and Spinopelvic Alignment in Patients Undergoing Lumbar Fusion: A Cross-Sectional Study
Source: J Clin Med. 2026 Jul 24;15(15):5812. doi: 10.3390/jcm15155812 (PMC13466865; doi:10.3390/jcm15155812)
Supplement: Supplementary file 1 [file jcm-15-05812-s001.zip › Supplementary_Material Table_S1.pdf]

Table S1. Correlation analysis between trunk isokinetic torques, paraspinal muscle degeneration, and spinopelvic alignment in the total population.

	Age	BMI	Gender	ODI	mismatch	LL	SS	PT	PI	PI–LL	ROT torque	E torque	F torque	LF torque	VAS	MF-fCSA	ES-fCSA	PS-fCSA	MF-FI	ES-FI	PS-FI
**Age**	1.000	-0.027	-0.164	0.187*	0.144	-0.029	0.097	0.065	0.128	0.134	-0.138	-0.181*	-0.226*	-0.160	0.220*	-0.251**	-0.239**	-0.240**	0.348***	0.196*	0.054
**BMI**	-0.027	1.000	-0.016	-0.102	-0.062	0.000	-0.006	0.076	0.040	-0.016	0.286**	0.171	0.216*	0.257**	-0.090	0.143	0.193*	0.199*	-0.120	-0.055	-0.058
**Gender**	-0.164	-0.016	1.000	-0.178	-0.157	-0.043	-0.078	-0.258**	-0.233*	-0.134	0.191*	0.168	0.242**	0.227*	-0.232*	0.572***	0.462***	0.779***	0.072	0.028	-0.205*
**ODI**	0.187*	-0.102	-0.178	1.000	0.126	-0.099	-0.013	0.112	0.087	0.165	-0.362***	-0.340***	-0.436***	-0.377***	0.461***	-0.185*	-0.231*	-0.202*	0.142	-0.101	0.082
**mismatch**	0.144	-0.062	-0.157	0.126	1.000	-0.540***	-0.196*	0.515***	0.389***	0.864***	-0.246**	-0.209*	-0.176	-0.225*	0.135	-0.315***	-0.161	-0.219*	-0.055	0.037	0.077
**LL**	-0.029	0.000	-0.043	-0.099	-0.540***	1.000	0.720***	-0.216*	0.291**	-0.627***	0.190*	0.143	0.053	0.171	0.158	0.230*	0.110	0.041	0.004	-0.053	0.009
**SS**	0.097	-0.006	-0.078	-0.013	-0.196*	0.720***	1.000	-0.239**	0.438***	-0.257**	0.167	0.101	-0.003	0.097	0.202*	0.113	0.055	-0.075	-0.091	-0.063	0.047
**PT**	0.065	0.076	-0.258**	0.112	0.515***	-0.216*	-0.239**	1.000	0.644***	0.710***	-0.235*	-0.242**	-0.262**	-0.140	0.110	-0.350***	-0.227*	-0.246**	-0.073	-0.029	0.030
**PI**	0.128	0.040	-0.233*	0.087	0.389***	0.291**	0.438***	0.644***	1.000	0.500***	-0.070	-0.126	-0.233*	-0.057	0.243**	-0.198*	-0.168	-0.253**	-0.055	-0.031	0.136
**PI–LL**	0.134	-0.016	-0.134	0.165	0.864***	-0.627***	-0.257**	0.710***	0.500***	1.000	-0.239**	-0.228*	-0.229*	-0.199*	0.083	-0.329***	-0.226*	-0.225*	-0.072	0.036	0.089
**ROT torque**	-0.138	0.286**	0.191*	-0.362***	-0.246**	0.190*	0.167	-0.235*	-0.070	-0.239**	1.000	0.727***	0.707***	0.728***	-0.106	0.368***	0.246**	0.227*	-0.162	-0.035	-0.137
**E torque**	-0.181*	0.171	0.168	-0.340***	-0.209*	0.143	0.101	-0.242**	-0.126	-0.228*	0.727***	1.000	0.766***	0.698***	-0.051	0.239**	0.261**	0.258**	-0.187*	-0.144	-0.095
**F torque**	-0.226*	0.216*	0.242**	-0.436***	-0.176	0.053	-0.003	-0.262**	-0.233*	-0.229*	0.707***	0.766***	1.000	0.790***	-0.215*	0.302***	0.294**	0.277**	-0.234*	-0.157	-0.088
**LF torque**	-0.160	0.257**	0.227*	-0.377***	-0.225*	0.171	0.097	-0.140	-0.057	-0.199*	0.728***	0.698***	0.790***	1.000	-0.215*	0.328***	0.333***	0.272**	-0.232*	-0.079	-0.058
**VAS**	0.220*	-0.090	-0.232*	0.461***	0.135	0.158	0.202*	0.110	0.243**	0.083	-0.106	-0.051	-0.215*	-0.215*	1.000	-0.181*	-0.237**	-0.192*	0.108	-0.201*	0.053
**MF-fCSA**	-0.251**	0.143	0.572***	-0.185*	-0.315***	0.230*	0.113	-0.350***	-0.198*	-0.329***	0.368***	0.239**	0.302***	0.328***	-0.181*	1.000	0.516***	0.561***	-0.018	0.101	-0.157
**ES-fCSA**	-0.239**	0.193*	0.462***	-0.231*	-0.161	0.110	0.055	-0.227*	-0.168	-0.226*	0.246**	0.261**	0.294**	0.333***	-0.237**	0.516***	1.000	0.483***	0.025	-0.012	-0.229*
**PS-fCSA**	-0.240**	0.199*	0.779***	-0.202*	-0.219*	0.041	-0.075	-0.246**	-0.253**	-0.225*	0.227*	0.258**	0.277**	0.272**	-0.192*	0.561***	0.483***	1.000	0.073	0.009	-0.349***
**MF-FI**	0.348***	-0.120	0.072	0.142	-0.055	0.004	-0.091	-0.073	-0.055	-0.072	-0.162	-0.187*	-0.234*	-0.232*	0.108	-0.018	0.025	0.073	1.000	0.499***	-0.043
**ES-FI**	0.196*	-0.055	0.028	-0.101	0.037	-0.053	-0.063	-0.029	-0.031	0.036	-0.035	-0.144	-0.157	-0.079	-0.201*	0.101	-0.012	0.009	0.499***	1.000	0.047
**PS-FI**	0.054	-0.058	-0.205*	0.082	0.077	0.009	0.047	0.030	0.136	0.089	-0.137	-0.095	-0.088	-0.058	0.053	-0.157	-0.229*	-0.349***	-0.043	0.047	1.000

Values are Spearman correlation coefficients. * $p < 0.05$; ** $p < 0.01$; *** $p < 0.001$. PI–LL mismatch is defined as PI–LL > 10°. Gender is coded as male = 1 and female = 0. The matrix presents correlation coefficients together with their statistical significance.

Abbreviations: BMI, body mass index; VAS, visual analogue scale; LL, lumbar lordosis; SS, sacral slope; PT, pelvic tilt; PI, pelvic incidence; PI–LL, pelvic incidence minus lumbar lordosis; PI–LL mismatch, PI–LL > 10°; ROT torque, rotation torque; E torque, extension torque; F torque, flexion torque; LF torque, lateral flexion torque; ODI, Oswestry Disability Index; MF-fCSA, multifidus functional cross-sectional area; ES-fCSA, erector spinae functional cross-sectional area; PS-fCSA, psoas functional cross-sectional area; MF-FI, multifidus fatty infiltration; ES-FI, erector spinae fatty infiltration; PS-FI, psoas fatty infiltration.
