# Supplementary material for: Associations Between Paraspinal Muscle Degeneration, Trunk Isokinetic Strengths, and Spinopelvic Alignment in Patients Undergoing Lumbar Fusion: A Cross-Sectional Study
Source: J Clin Med. 2026 Jul 24;15(15):5812. doi: 10.3390/jcm15155812 (PMC13466865; doi:10.3390/jcm15155812)
Supplement: Supplementary file 1 [file jcm-15-05812-s001.zip › Supplementary_Material Table_S2.pdf]

Table S2. Correlation analysis between trunk isokinetic torques, paraspinal muscle degeneration, and spinopelvic alignment in males.

	Age	BMI	ODI	mismatch	LL	SS	PT	PI	PI–LL	ROT torque	E torque	F torque	LF torque	VAS	MF-fCSA	ES-fCSA	PS-fCSA	MF-FI	ES-FI	PS-FI
**Age**	1.000	-0.128	0.120	0.242	-0.185	-0.045	0.045	0.055	0.190	0.024	-0.038	-0.112	-0.164	0.245	-0.300*	-0.319*	-0.401**	0.251	0.171	0.117
**BMI**	-0.128	1.000	-0.183	0.076	-0.009	-0.150	0.262*	0.181	0.123	0.312*	0.198	0.282*	0.351**	-0.078	0.282*	0.248	0.371**	-0.098	-0.077	-0.074
**ODI**	0.120	-0.183	1.000	0.168	-0.194	-0.183	0.184	0.017	0.223	-0.339**	-0.269*	-0.398**	-0.288*	0.420**	-0.067	-0.109	-0.163	0.166	-0.028	0.138
**mismatch**	0.242	0.076	0.168	1.000	-0.715***	-0.264*	0.468***	0.414**	0.863***	-0.244	-0.223	-0.182	-0.229	0.263*	-0.330*	-0.026	-0.032	0.026	0.083	-0.026
**LL**	-0.185	-0.009	-0.194	-0.715***	1.000	0.585***	-0.294*	0.026	-0.729***	0.313*	0.265*	0.240	0.332*	-0.033	0.370**	0.206	0.111	-0.035	-0.165	-0.163
**SS**	-0.045	-0.150	-0.183	-0.264*	0.585***	1.000	-0.420**	0.170	-0.278*	0.275*	0.207	0.139	0.243	-0.073	0.265*	0.255	0.067	-0.150	-0.087	-0.120
**PT**	0.045	0.262*	0.184	0.468***	-0.294*	-0.420**	1.000	0.706***	0.693***	-0.272*	-0.316*	-0.254	-0.080	0.145	-0.261*	-0.119	-0.035	-0.044	0.019	-0.003
**PI**	0.055	0.181	0.017	0.414**	0.026	0.170	0.706***	1.000	0.599***	-0.063	-0.165	-0.097	0.066	0.055	-0.058	0.006	-0.088	-0.059	0.033	0.011
**PI–LL**	0.190	0.123	0.223	0.863***	-0.729***	-0.278*	0.693***	0.599***	1.000	-0.267*	-0.303*	-0.258	-0.192	0.140	-0.318*	-0.143	-0.084	-0.060	0.134	0.068
**ROT torque**	0.024	0.312*	-0.339**	-0.244	0.313*	0.275*	-0.272*	-0.063	-0.267*	1.000	0.647***	0.721***	0.728***	-0.019	0.429***	0.180	0.219	0.008	0.019	-0.246
**E torque**	-0.038	0.198	-0.269*	-0.223	0.265*	0.207	-0.316*	-0.165	-0.303*	0.647***	1.000	0.776***	0.752***	-0.053	0.216	0.262*	0.226	-0.087	-0.102	-0.143
**F torque**	-0.112	0.282*	-0.398**	-0.182	0.240	0.139	-0.254	-0.097	-0.258	0.721***	0.776***	1.000	0.809***	-0.082	0.275*	0.191	0.205	-0.195	-0.234	-0.206
**LF torque**	-0.164	0.351**	-0.288*	-0.229	0.332*	0.243	-0.080	0.066	-0.192	0.728***	0.752***	0.809***	1.000	-0.015	0.371**	0.328*	0.304*	-0.252	-0.132	-0.103
**VAS**	0.245	-0.078	0.420**	0.263*	-0.033	-0.073	0.145	0.055	0.140	-0.019	-0.053	-0.082	-0.015	1.000	-0.117	-0.220	0.019	0.121	-0.039	0.020
**MF-fCSA**	-0.300*	0.282*	-0.067	-0.330*	0.370**	0.265*	-0.261*	-0.058	-0.318*	0.429***	0.216	0.275*	0.371**	-0.117	1.000	0.502***	0.299*	-0.012	0.110	0.112
**ES-fCSA**	-0.319*	0.248	-0.109	-0.026	0.206	0.255	-0.119	0.006	-0.143	0.180	0.262*	0.191	0.328*	-0.220	0.502***	1.000	0.371**	-0.132	-0.158	-0.127
**PS-fCSA**	-0.401**	0.371**	-0.163	-0.032	0.111	0.067	-0.035	-0.088	-0.084	0.219	0.226	0.205	0.304*	0.019	0.299*	0.371**	1.000	-0.012	-0.142	-0.280*
**MF-FI**	0.251	-0.098	0.166	0.026	-0.035	-0.150	-0.044	-0.059	-0.060	0.008	-0.087	-0.195	-0.252	0.121	-0.012	-0.132	-0.012	1.000	0.442***	-0.156
**ES-FI**	0.171	-0.077	-0.028	0.083	-0.165	-0.087	0.019	0.033	0.134	0.019	-0.102	-0.234	-0.132	-0.039	0.110	-0.158	-0.142	0.442***	1.000	0.107
**PS-FI**	0.117	-0.074	0.138	-0.026	-0.163	-0.120	-0.003	0.011	0.068	-0.246	-0.143	-0.206	-0.103	0.020	0.112	-0.127	-0.280*	-0.156	0.107	1.000

Values are Spearman correlation coefficients. * $p < 0.05$; ** $p < 0.01$; *** $p < 0.001$. PI–LL mismatch is defined as PI–LL > 10°. The matrix presents correlation coefficients together with their statistical significance.

Abbreviations: BMI, body mass index; VAS, visual analogue scale; LL, lumbar lordosis; SS, sacral slope; PT, pelvic tilt; PI, pelvic incidence; PI–LL, pelvic incidence minus lumbar lordosis; PI–LL mismatch, PI–LL > 10°; ROT torque, rotation torque; E torque, extension torque; F torque, flexion torque; LF torque, lateral flexion torque; ODI, Oswestry Disability Index; MF-fCSA, multifidus functional cross-sectional area; ES-fCSA, erector spinae functional cross-sectional area; PS-fCSA, psoas functional cross-sectional area; MF-FI, multifidus fatty infiltration; ES-FI, erector spinae fatty infiltration; PS-FI, psoas fatty infiltration.
